# Supplementary material for: Resolving the full spectrum of human genome variation using Linked-Reads
Source: Genome Res. 2019 Apr;29(4):635–45. doi: 10.1101/gr.234443.118 (PMC6442396; doi:10.1101/gr.234443.118)
Supplement: Supplemental Material [file supp_gr.234443.118_Supplemental_longranger-2.2.zip › longranger-2.2.tar.bz2!/lr2.2-public/lib/stan/cmdstan/stan/doc/api/html/sub_8hpp_source.html]

Stan: src/stan/math/functions/sub.hpp Source File


|  |  |
| --- | --- |
|  | Stan  2.5.0  probability, sampling & optimization |


- Main Page
- Related Pages
- Namespaces
- Classes
- Files

- File List
- File Members

All Classes Namespaces Files Functions Variables Typedefs Enumerations Enumerator Friends Macros Pages

- src
- stan
- math
- functions

sub.hpp

Go to the documentation of this file.

1 #ifndef STAN\_\_MATH\_\_FUNCTIONS\_\_SUB\_HPP

2 #define STAN\_\_MATH\_\_FUNCTIONS\_\_SUB\_HPP

3

4 #include <vector>

5 #include <cstddef>

6

7 namespace stan {

8  namespace math {

9

10  inline void sub(std::vector<double>& x, std::vector<double>& y,

11  std::vector<double>& result) {

12  result.resize(x.size());

13  for (size\_t i = 0; i < x.size(); ++i)

14  result[i] = x[i] - y[i];

15  }

16

17  }

18 }

19

20 #endif

stan::math::sub

void sub(std::vector< double > &x, std::vector< double > &y, std::vector< double > &result)

**Definition:** sub.hpp:10


---

[ Stan Home Page ]

*© 2011–2014,
Stan Development Team.*
